# High *BECN1* Expression Negatively Correlates with *BCL2* Expression and Predicts Better Prognosis in Diffuse Large B-Cell Lymphoma: Role of Autophagy

**DOI:** 10.3390/cells12151924

**Published:** 2023-07-25

**Authors:** Amreen Salwa, Alessandra Ferraresi, Eleonora Secomandi, Letizia Vallino, Riccardo Moia, Andrea Patriarca, Beatrice Garavaglia, Gianluca Gaidano, Ciro Isidoro

**Affiliations:** 1 Laboratory of Molecular Pathology, Department of Health Sciences, Università del Piemonte Orientale, Via P. Solaroli 17, 28100 Novara, Italy; salwa.amreen@uniupo.it (A.S.); alessandra.ferraresi@med.uniupo.it (A.F.); eleonora.secomandi@uniupo.it (E.S.); letizia.vallino@uniupo.it (L.V.); beatrice.garavaglia@uniupo.it (B.G.); 2 Division of Hematology, Department of Translational Medicine, Università del Piemonte Orientale, Via P. Solaroli 17, 28100 Novara, Italy; riccardo.moia@uniupo.it (R.M.); andrea.patriarca@uniupo.it (A.P.)

**Keywords:** lymphoma, autophagy, BECLIN-1, BCL-2, venetoclax, apoptosis, chemoresistance, overall survival, TCGA, prognosis, chemotherapy

## Abstract

Diffuse large B-cell lymphoma (DLBCL) is characterized by high molecular and clinical heterogeneity. Autophagy, a lysosome-driven catabolic process devoted to macromolecular turnover, is fundamental in maintaining normal hematopoietic stem cells and progenitors homeostasis, and its dysregulation plays a critical role in the initiation and progression of hematological malignancies. One main regulator of autophagy is BECLIN-1, which may interact alternatively with either BCL-2, thus allowing apoptosis, or PI3KC3, thus promoting autophagy. The altered expression of *BCL2* and *BECN1* correlates with lymphoma outcomes, but whether this is associated with dysregulated cross-talk between autophagy and apoptosis remains to be elucidated. Analysis of the TCGA database revealed that *BCL2* and *BECN1* mRNA expression were inversely correlated in DLBCL patients. In representative DLBCL cell lines exposed to doxorubicin, the cells highly expressing BCL-2 were resistant, while the ones highly expressing BECLIN-1 were sensitive, and this correlated with low and high autophagy flux, respectively. Venetoclax targeting of BCL-2 increased while the spautin-1-mediated inhibition of BECLIN-1-dependent autophagy reversed doxorubicin sensitivity in the former and in the latter, respectively. By interrogating the TCGA DLBCL dataset, we found that *BCL2* and *BECN1* acted as negative and positive prognostic markers for DLBCL, respectively. The differentially expressed gene analysis in the respective cohorts revealed that *BCL2* positively correlated with oncogenic pathways (e.g., glucose transport, HIF1A signaling, JAK-STAT signaling, PI3K-AKT-mTOR pathway) and negatively correlated with autophagy-related transcripts, while *BECN1* showed the opposite trend. Notably, patients with high *BECN1* expression displayed longer survival. Our data reveal, for the first time, that the modulation of BECLIN-1-dependent autophagy influences the prognosis of DLBCL patients and provide a mechanistic explanation supporting the therapeutic use of drugs that, by stimulating autophagy, can sensitize lymphoma cells to chemotherapy.

## 1. Introduction

Diffuse large B-cell lymphoma (DLBCL) is the most common subtype of B-cell non-Hodgkin lymphoma (B-NHL) in adults, accounting for 30–40% of B-NHL cases globally [1,2,3]. DLBCL is characterized by the clonal proliferation of mature B-cells expressing the B-cell surface antigens CD19, CD20, CD22, and CD79a/b. DLBCL presents high clinical heterogeneity that reflects the molecular heterogeneity of gene expression [3,4,5].

Although the majority (60–70%) of DLBCL patients are conceivably cured by rituximab plus cyclophosphamide, doxorubicin, vincristine, and prednisone (R-CHOP) treatment, the remaining patients relapse or show refractoriness and still represent an unmet clinical need [3]. Therefore, the identification at the time of diagnosis of molecular characteristics that can anticipate disease progression and chemotherapy responses is fundamental.

Based on the cell of origin (COO), DLBCL is classified as germinal center B-cell (GCB) (40–50%) or activated B-cell (ABC) (50–60%). A fraction of DLBCL patients (10–15%) remain unclassified. GCB lymphoma cases show a better clinical outcome than ABC cases, when treated with R-CHOP [3,4,6]. In addition to COO, chromosomal translocations of *BCL6*, *BCL2*, and *c-MYC* are common cytogenetic abnormalities in DLBCL, and GCB lymphomas harboring these genetic lesions are called “double hit” lymphoma (*c-MYC* plus *BCL2* or *BCL6*) or triple hit lymphoma (*c-MYC* plus *BCL2* plus *BCL6*), respectively [3,6,7,8].

BCL-2 is an anti-apoptotic protein, belonging to the Bcl-2 family, regulating apoptosis through the interaction and inactivation of pro-apoptotic proteins [9]. *BCL2*-inactivating mutations are very rare in DLBCL [10]. *BCL2* translocations are found in 20–30% of de novo DLBCL, and most cases are observed in the GCB subtype [11,12]. Additionally, *BCL2* amplification has been reported in DLBCL [13,14]. *BCL2* abnormal expression dysregulates apoptosis and results in a survival advantage for affected B-cells [9,15,16], thus worsening the clinical outcome in DLBCL patients [9,15]. Consistently, inhibition of the Bcl-2 family proteins can enhance chemosensitivity in B-cell lymphoma [9,15].

Autophagy is a lysosomal-dependent catabolic process for macromolecular and organelle degradation that has a pivotal role in hematopoietic stem cells homeostasis, and it is dysregulated in blood cancers [17,18]. In hematologic malignancies, autophagy plays a dual role, either supporting cancer cell survival towards chemoresistance or exerting tumor-suppressive functions [19].

BECLIN-1, the first identified mammalian autophagy gene product, was originally isolated as a BCL-2-interacting protein [20]. BECLIN-1 triggers the autophagy machinery by interacting either with BCL-2 or class III PI3K via its BH3 domain, and thus it plays a crucial role in the regulation of cell fate between cell death and autophagy [21]. *BECN1* is regarded as a haploinsufficient tumor suppressor gene since its heterozygous deletion in transgenic mice favors the spontaneous development of tumors in different tissues [22]. In particular, *Becn1*^+/−^ mice show a higher frequency of B-cell lymphomas [22,23]. Notably, we and others reported that the high expression of BECLIN-1 predicts better prognosis in B-NHL patients [24,25,26].

In this work, we investigated the clinical relevance of BECLIN-1-mediated autophagy and of its mechanistic association with the pro-tumorigenic role of BCL-2 in DLBCL progression and the response to chemotherapy. We tested the hypothesis that promoting BECLIN-1-mediated autophagy could improve the clinical outcome in DLBCL patients. On interrogation of the Cancer Genome Atlas (TCGA) transcriptome of DLBCL patients, we found that *BCL2* and *BECN1* mRNA expression were inversely correlated. In vitro experiments with three DLBCL cell lines representative of different genetic backgrounds showed that cells with high BCL-2 levels were resistant, whereas cells with high BECLIN-1-dependent autophagy were sensitive to doxorubicin.

Targeting BCL-2 with venetoclax restored the autophagy flux and the sensitivity to doxorubicin-induced cell death, and, conversely, spautin-1-mediated inhibition of BECLIN-1-dependent autophagy decreased the sensitivity to the chemotherapeutic. Consistent with the positive role of autophagy, patients bearing DLBCL with high *BECN1* expression display longer overall survival.

Our data support BECLIN-1 as a valuable prognostic biomarker to predict the response to chemotherapy and additionally indicate BECLIN-1-dependent autophagy as a target pathway to improve the response to chemotherapy and the clinical outcome in DLBCL patients.

## 2. Materials and Methods

### 2.1. Cell Culture

RI-1, OCI-LY8, and SUDHL-8 human DLBCL cells lines with different genetic backgrounds for relevant genes (Table 1) were purchased from the German Collection of Microorganisms and Cell Cultures (DSMZ GmbH; Braunschweig, Germany).

RI-1 and SUDHL-8 cells were cultured in RPMI 1640 medium (cod. R8758; Sigma Aldrich, St. Louis, MO, USA) supplemented with 10% heated-inactivated FBS (cod. ECS0180L; Euroclone, Milan, Italy), 1% glutamine (cod. G7513; Sigma Aldrich, St. Louis, MO, USA), and 1% penicillin/streptomycin (cod. P0781; Sigma Aldrich, St. Louis, MO, USA).

OCI-LY8 cells were cultured in Minimum Essential Medium (MEM) (cod. M2279; Sigma-Aldrich, St. Louis, MO, USA) supplemented with 10% heat-inactivated FBS (cod. ECS0180L; Euroclone, Milan, Italy), 1% glutamine (cod. G7513; Sigma-Aldrich, St. Louis, MO, USA), 1% non-essential amino acids (cod. M7145; Sigma-Aldrich, St. Louis, MO, USA), and 1% PES (cod. P0781; Sigma-Aldrich, St. Louis, MO, USA).

All the cell lines were maintained under standard culture conditions (37 °C, 5% CO_2_).

### 2.2. Reagents

Doxorubicin (Doxo, cod. 44583-1MG, Sigma-Aldrich, St. Louis, MO, USA) was dissolved in sterile water and used at a final concentration of 1 µM. Spautin-1 (Sp-1, Cod. SML0440-5MG, Sigma-Aldrich, St. Louis, MO, USA), a small molecule that inhibits autophagy by directing the proteasome degradation of BECLIN-1 [27], was dissolved in sterile water and used at a final concentration of 10 µM. Venetoclax (Ven, cod. ABT-199, GDC-0199-5MG, Selleckchem, Houston, TX, USA), a BH3-mimetic that targets the antiapoptotic BCL-2 protein [15], was dissolved in DMSO and used at a final concentration of 1 nM.

### 2.3. Antibodies

The following primary antibodies were employed for either immunofluorescence or Western blotting: mouse anti-LC3 (1:100 for IF, cod. 0231-100; Nanotools, Teningen, Germany), rabbit anti-LC3 (1:1000 for WB, cod. L7543; Sigma Aldrich, St. Louis, MO, USA), mouse anti-BECLIN-1 (1:500 for WB, cod. 612112; BD Biosciences, Franklin Lakes, NJ, USA), rabbit anti-BECLIN-1 (1:100 for IF, cod. PA5-86090, Invitrogen, Waltham, MA, USA), anti-p62 (1:500; cod. 8025; Cell Signaling, Danvers, MA, USA), mouse anti-BCL-2 (1:500 for WB, 1:100 for IF, cod. 15071; Cell Signaling Technologies, Danvers, MA, USA), rabbit anti-BAX (1:500 for WB, 1:50 for IF, cod. 2772: Cell Signaling Technologies, Danvers, MA, USA), mouse anti-caspase 3 (1:1000, cod. 9668; Cell Signaling Technologies, Danvers, MA, USA), rabbit anti-cleaved caspase 3 (1:1000, cod. 9661; Cell Signaling Technologies, Danvers, MA, USA), mouse anti-β-actin (1:2000, cod. A5441; Sigma Aldrich, St. Louis, MO, USA), mouse anti-histone H3 (1:500, cod. 61475; Active motif, Carlsbad, San Diego, CA, USA), and mouse anti-β-tubulin (1:1000, cod. T5201; Sigma Aldrich, St. Louis, MO, USA). The following were used as secondary antibodies for Western blotting: horseradish peroxidase (HRP)-conjugated goat anti-mouse (1:10,000; cod. 170-6516, BioRad, Hercules, CA, USA) or goat anti-rabbit (1:10,000; cod. 170-6515, BioRad, Hercules, CA, USA). The following secondary antibodies were used for immunofluorescence: AlexaFluor488-conjugated goat-anti-rabbit IgG antibody (1:1000; cod. A32731, Thermo Fisher Scientific, Waltham, MA, USA) or AlexaFluor555-conjugated goat-anti-mouse IgG antibody (1:1000; cod. A32727, Thermo Fisher Scientific, Waltham, MA, USA).

### 2.4. Western Blotting

Cells were seeded in T25 flasks and treated when confluence reached approximately 80%. A standard procedure was used to prepare cell homogenates, SDS-PAGE and blotting onto a PVDF filter (cod. 162-0177; BioRad, Hercules, CA, USA), and the saturation of the membrane with non-fat dry milk, as detailed in [28]. Filters were incubated with the appropriate primary antibodies overnight at 4 °C, followed by incubation with secondary HRP-conjugated antibodies for 1 h at room temperature. The bands were detected with Enhanced Chemiluminescence reagents (ECL, cod. NEL105001EA; Perkin Elmer, Waltham, MA, USA) and imaged with the VersaDOC Imaging System (Universal Hood II—S.N. 76S/04219; Biorad, Hercules, CA, USA). For loading control, the filters were re-probed with β-tubulin, β-actin, or histone H3. Densitometric analysis was performed with the Quantity One software (v. 4.5). 

### 2.5. Immunofluorescence

Cells were plated in T25 flasks and treated depending on the experiment performed. At the end of the treatment, the cells were cyto-spotted on glasses. Samples were processed for immunofluorescence as detailed in [28]. Briefly, the samples were incubated overnight at 4 °C with specific primary antibodies and, on the next day, incubated with dye-conjugated secondary antibodies for 1 h at room temperature. Nuclei were stained with the UV fluorescent dye 4′,6-diamidino-2-phenylindole (DAPI). Coverslips were mounted onto glasses using SlowFade reagent and imaged under a fluorescence microscope (Leica Microsystems, Wetzlar, Germany; DMI6000).

### 2.6. Cell Counting and Cell Cycle Analysis

Cells were seeded in T25 flasks and treated as appropriate depending on the experiment performed. At the end of the treatment, cells were collected, and an aliquot of cell suspension was diluted 1:1 with Trypan blue, which allows discrimination between living cells and dead cells. Cell counting was performed in triplicate for each experimental condition. Cells were fixed in 70% ice-cold ethanol and stored at −20 °C till the start of the cytofluorometric analysis. Cells were treated with RNAse for 30 min at 37 °C and stained with propidium iodide (PI, 50 µg/mL; cod. P4170, Sigma Aldrich, St. Louis, MO, USA) and acquired using a FACSCalibur (Becton Dickinson, Eysins, Switzerland) flow cytometer. For each sample, a fraction of 5000 events was analyzed. The analysis of the cytofluorimetric data obtained was performed using the Flowing software (v. 2.5.1)

### 2.7. Image Acquisition

Fluorescence images were acquired using a fluorescence microscope (Leica Microsystems, Wetzlar, Germany; DMI6000). For each experimental condition, at least three slides were prepared in separate experiments, and five to ten microscopic fields randomly chosen were imaged by two independent investigators unaware of the treatment. Quantification of fluorescence intensity was performed with the software ImageJ (v. 1.48). Representative images of selected fields are shown.

### 2.8. Statistical Analysis of Experimental Data

Statistical analysis was performed using the GraphPad Prism 5.0 software. Bonferroni’s multiple comparison test after one-way ANOVA analysis (unpaired, two-tailed) was employed. Significance was considered as follows: **** *p* < 0.0001; *** *p* < 0.001; ** *p* < 0.01; * *p* < 0.05; not significant (ns) *p* > 0.05. All experiments were reproduced at least three times in separate and independent replicates. All data are reported as the average ± S.D.

### 2.9. TCGA Database

Clinical and gene expression data were retrieved from the TCGA data repository (www.cBioportal.org, last accessed on 26 June 2023). The TCGA gene expression profile was measured using the Illumina HiSeq 2000 RNA sequencing platform (Illumina Inc., 9885 Towne Centre Drive, San Diego, CA 92121, USA). The RNA-Seq by Expectation-Maximization (RSEM) normalized count was used to estimate gene level expression. Gene variables were measured by median absolute deviation.

The DLBCL dataset accounts for 48 patients (TCGA, Firehose Legacy) and RNA-seq and clinical data are available in the repository (https://cancergenome.nih.gov or www.cBioportal.org, last accessed on 26 June 2023). This cohort of DLBCL patients included 22 males and 26 females, and the median age was 57 years (range: 23–82). The correlation with patient tumor stage was determined: 7 (35.4%) were classified as advanced stage (III/IV), 25 (52.1%) were classified as stage II/I, and, for 6 (12.5%) patients, the status was not available (N/A). Information regarding the therapy administered to the patients was not included on the TCGA portal. Clinical outcomes were classified as a complete or partial response to therapy.

### 2.10. Statistical Analysis of Gene Expression and Clinical Outcome

Kaplan–Meier curves, correlation studies, biological processes, and heatmaps were obtained by focusing on the TCGA cohort of DLBCL patients (N = 48). For statistical significance, the analysis was focused on tumors with the most representative mRNA expression.

*BCL2* and *BECN1* mRNA expression was sub-classified into high and low expression groups based on the level of mRNA expression (z-score values). Low versus high mRNA expression was defined relative to the median expression level of all patients in the form of a box plot and was used to investigate the relationship between the dichotomized groups of *BCL2* and *BECN1* expression. To reduce the potential bias from dichotomization, the mRNA expression levels of *BCL2* and *BECN1* were compared based on high and low expression groups using a *t*-test (Welch two-sample *t*-test). All cut-off values were set before the analysis, and all the tests were two-tailed [29].

Survival analysis was performed using the SAS software for the following genes: *BCL2* and *BECN1* based on the level of mRNA expression (high vs. low expression group). Survival curves of these two groups were estimated from the Kaplan–Meier plots and compared using the Cox regression model, assuming an ordered trend for the two groups, as described previously. The log-rank test was used to determine statistical significance. A *p*-value < 0.05 was considered significant.

All statistical analyses were performed using the R (3.6.1 version, The R Foundation for Statistical Computing, Vienna, Austria) and SAS software (9.4. version, SAS Institute Inc., Cary, NC, USA).

### 2.11. Correlation Analysis and Screening of DEGs in Correlation with BCL2 and BECN1 Gene Expression

Scatterplots were employed to represent the correlations between the expression of relevant biomarkers in the patient cohort, as previously reported [30]. Pearson’s correlation analyses were performed to identify the correlation between the *BECN1* and *BCL2* genes. Regression was estimated by calculating Pearson’s correlation coefficients (r) and the relative *p*-values.

TBtools (https://github.com/CJ-Chen/TBtools/, accessed on 25 March 2023) was used to identify the DEGs in correlation with *BECN1* and *BCL2*. To identify the DEGs, cut-off criteria were set based on Spearman’s correlation values, i.e., correlation coefficient value greater than +0.40 (positively correlated) or lower than −0.40 (negatively correlated) and *p*-value < 0.001 (−log10 (*p*-value) threshold was fixed above 2.0). DEGs are represented in the form of Volcano plots.

### 2.12. Gene Ontology and Pathway Enrichment Analysis of DEGs

The DAVID bioinformatics functional annotation tool (https://david.ncifcrf.gov/summary.jsp, accessed on 10 March 2023) was used to analyze Gene Ontology (GO) biological processes, and Kyoto Encyclopedia of Genes and Genomes (KEGG) pathways were obtained with the help of positive and negative DEGs. Data are presented in bar graphs displaying the number of transcripts belonging to each positively and negatively associated biological process.

Based on the different expression values, MeV4 (http://mev.tm4.org/, accessed on 25 March 2023), a freely available software application, was used to create heatmaps.

## 3. Results

### 3.1. Oncoprint of Somatic Mutations in BCL2 and BECN1 Genes in DLBCL Patients

**Figure 1:**
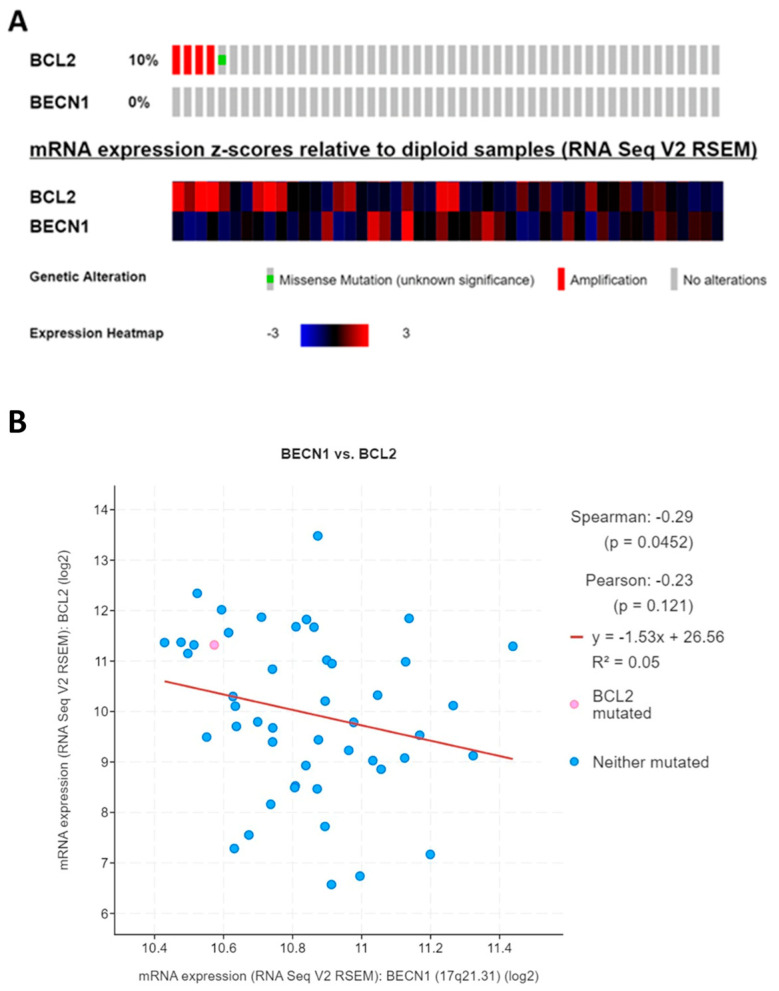
Oncoprint of somatic mutation in *BCL2* and *BECN1* genes and their correlation analysis. (**A**) The oncoprint shows the genetic alterations and mRNA expression levels of *BCL2* and *BECN1* in 48 lymphoid neoplasm diffuse large B-cell lymphoma patients (TCGA, Firehose Legacy). The heatmap reports in red and blue the upregulated and downregulated genes, respectively. (**B**) Scatterplots showing the negative correlation between *BECN1* and *BCL2*.

We interrogated the DLBCL dataset (Firehose Legacy) available in the TCGA bioportal to monitor the expression and mutation profiles of the *BCL2* and *BECN1* genes. As represented in Figure 1A, out of 48 DLBCL cases, *BCL2* was amplified in four patients and a missense mutation was found in one patient. This was consistent with the literature reporting that a small percentage of DLBCL patients bear a mutation in the *BCL2* gene [10]. On the other hand, the transcriptomic analysis shown in the heatmap confirmed that most of the patients displayed high levels of *BCL2* mRNA, which could arise from gene translocation [11,12] or gene amplification [13,14]. On the contrary, *BECN1* was not affected by gene mutation or chromosomal alteration. The heatmap below depicts that the majority of DLBCL patients displayed high levels of *BCL2*, while the expression of *BECN1* was low or very low in most of them. Next, we evaluated the relationship between the expression of the two genes. The scatterplot reported in Figure 1B shows that the mRNA expression of *BECN1* was significantly negatively correlated with that of *BCL2* (*p* = 0.0452).

### 3.2. Differential BECLIN-1/BCL-2 Expression Defines the Autophagy Flux and the Sensitivity of DLBCL Cells to Doxorubicin

Based on previous reports by our group and others [24,25,26], we hypothesized that BECLIN-1-dependent autophagy may sensitize DLBCL patients to chemotherapy and that this outcome is correlated with BCL-2 downregulation. To experimentally prove this, we chose three DLBCL cell lines with different genetic backgrounds, representing the heterogeneity of BECLIN-1 and BCL-2 expression (see Table 1 in Materials and Methods), to address whether and how autophagy modulation can impact the chemotherapy response.

**Figure 2:**
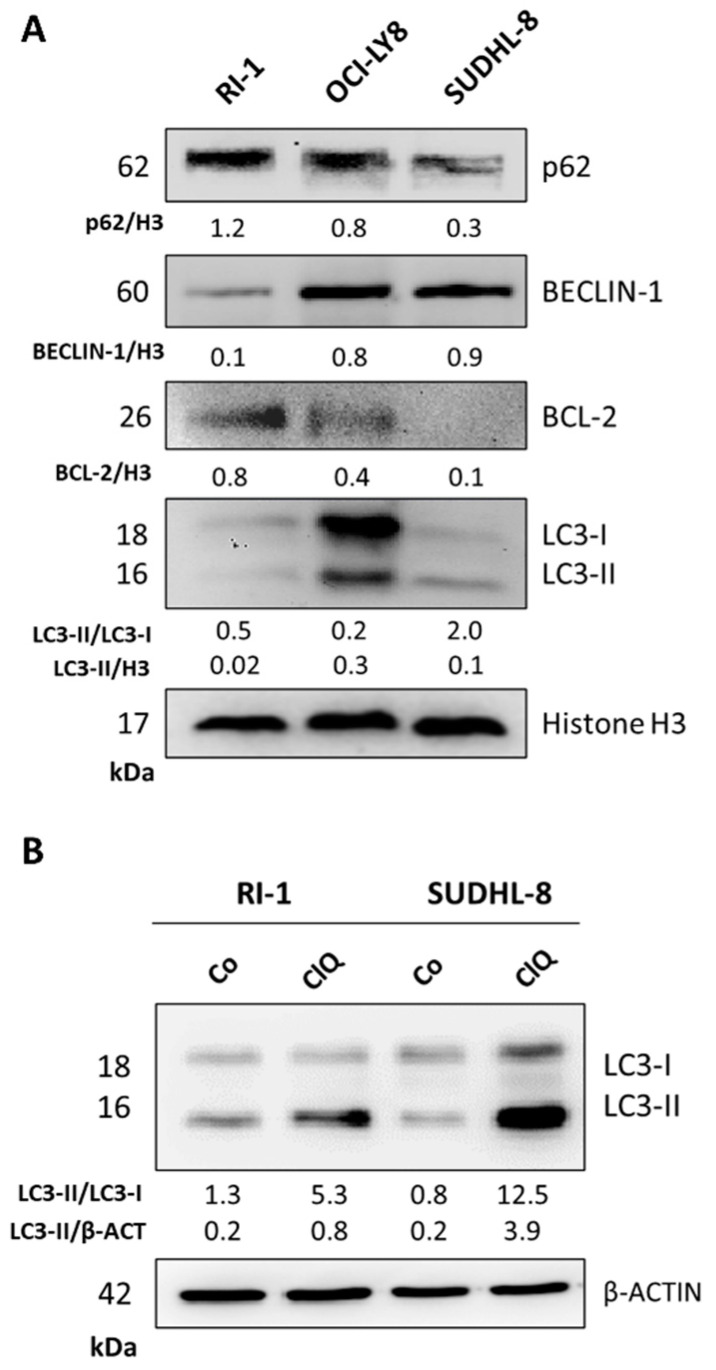
Basal levels of BECLIN-1, BCL-2 and LC3 in three representative DLBCL cell lines. Three cell lines (RI-1, OCI-LY8, SUDHL-8) representative of the heterogeneity of DLBCL cells were analyzed by Western blotting for the expression of the autophagy markers p62/SQSTM1 and LC3, the pro-autophagic oncosuppressor protein BECLIN-1 and the pro-apoptotic oncogenic protein BCL-2. Histone H3 and β-actin were chosen as housekeeping proteins for loading controls. In panel (**A**), the basal level of protein expression is indicated. In panel (**B**), the conversion of LC3-I into LC3-II and the accumulation of the latter in cells exposed or not to 30 µM chloroquine, an inhibitor of autophagosome degradation, are shown. Densitometric data are reported. The Western blotting results shown are representative of triplicates with a similar trend of protein expression.

As shown by Western blotting in Figure 2A, BCL-2 was highly expressed in RI-1 and barely detectable in SUDHL-8 cells, while BECLIN-1 expression showed the opposite trend, so that the BECLIN-1/BCL-2 ratio was 0.125 in the former and 10 in the later, while OCI-LY8 cells presented a 1:1 ratio. We then assayed the expression of autophagy markers. P62/SQSTM1 monitors cargo degradation and is assumed as a readout of autophagy flux [31]. The conversion of LC3-I into LC3-II is an indirect measure of autophagosome formation, while the level of LC3-II indirectly mirrors the amount of autophagosome that accumulates in the cell [31]. SUDHL-8 showed the lowest level of p62 along with the highest ratio of LC3-I to LC3-II conversion and relatively low accumulation of LC3-II. In contrast, RI-1 cells presented the largest accumulation of p62, similar to the levels of LC3-II. This indicates that autophagosome formation and autophagosome consumption (i.e., autophagy flux) are much higher in SUDHL-8 cells compared to RI-1 cells. Based on the p62 and LC3-I and LC3-II levels, OCI-LY8 cells showed an intermediate level of autophagy flux, while showing a relative inability to form autophagosomes (as indicated by the relatively low rate of LC3-I to LC3-II conversion). We treated the cells with chloroquine [31] to better characterize the autophagosome formation and the autophagy flux in RI-1 and SUDHL-8 cells. The data in Figure 2B can be interpreted as indicating the SUDHL-8 cells as the ones with the greatest production of autophagosomes and the highest level of autophagy flux, as demonstrated by the amount of LC3-II accumulated when the fusion of the autophagosome with the lysosome was inhibited by chloroquine [31].

**Figure 3:**
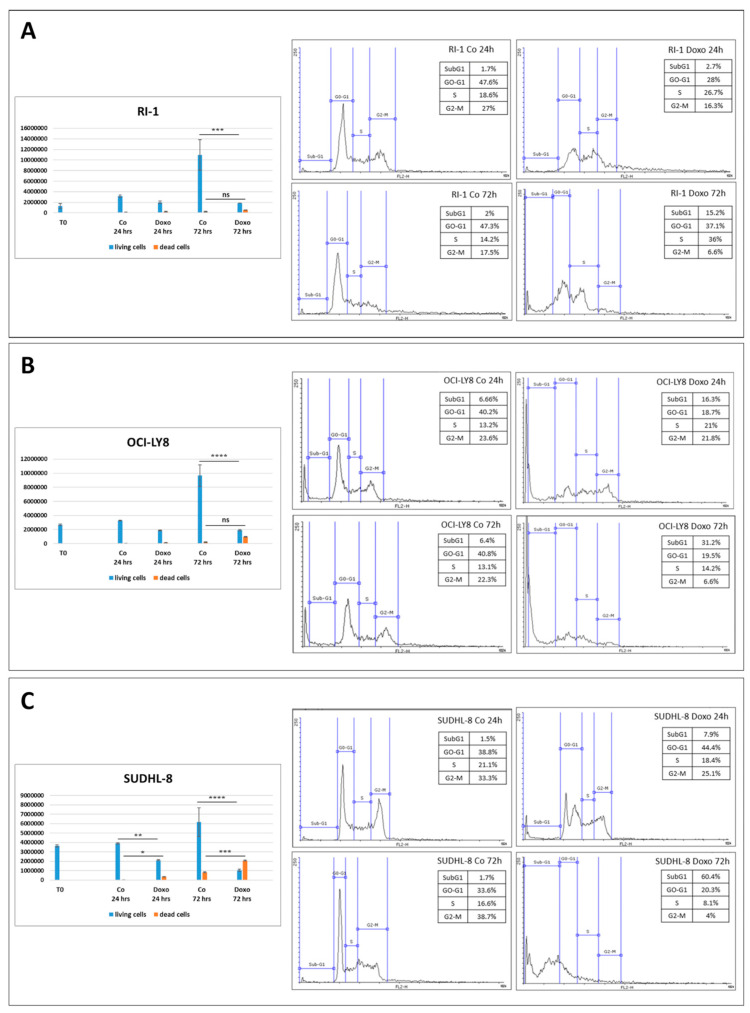
Evaluation of doxorubicin-induced cell toxicity. RI-1 (**A**), OCI-LY8 (**B**) and SUDHL-8 cells (**C**) were exposed to doxorubicin for 24 h and 72 h. Cell counting by Trypan blue exclusion was performed in triplicate. Cell cycle analysis was performed using a FacScan flow cytometer. Statistical analysis was performed using GraphPad Prism 8.0 software. Bonferroni’s multiple comparison test after one-way ANOVA analysis (unpaired, two-tailed) was employed. Significance was considered as follows: **** *p* < 0.0001; *** *p* < 0.001; ** *p* < 0.01; * *p* < 0.05; ns *p* > 0.05.

Next, we tested the chemosensitivity of the three cell lines to doxorubicin, monitoring cell growth and cell death at two time points (i.e., 24 h and 72 h) by cell counting and cytofluorometric cell cycle analysis (Figure 3). RI-1 cells challenged with doxorubicin displayed cytostatic effects rather than cytotoxicity. Indeed, the number of dead cells after doxorubicin treatment was negligible and, consistently, the cell cycle analysis showed that the SubG1 peaks (2.7% and 15.2% at 24 h and 72 h time points, respectively) were very modest, while the predominant effect was cell cycle arrest in the S phase (26.7% and 36% at 24 h and 72 h time points, respectively) (Figure 3A). In doxorubicin-treated OCI-LY8 cells cytotoxicity appeared more evident. These cells were more likely to undergo cell death (SubG1 = 16.3% and 31.2% at 24 h and 72 h time points, respectively), and after 72 h they displayed a strong reduction in the G2-M phase compared to the controls (6.6% vs. 23.6%) (Figure 3B). In doxorubicin-treated SUDHL-8 cells, a remarkable increase in the number of dead cells was observed with the incubation time. Moreover, the cytofluorometric analysis revealed that doxorubicin-treated cells displayed a much greater increase in cell death (SubG1 = 7.9% and 60.4% at 24 h and 72 h time points, respectively) compared to the other two cell lines (Figure 3C).

These data indicate that RI-1 cells are the least and SUDHL-8 cells are the most sensitive to doxorubicin. To corroborate the above findings, we monitored the expression of two important effectors in the regulation of apoptosis, BCL-2 and BAX, with anti-apoptotic and pro-apoptotic activity, respectively. Additionally, we evaluated whether and how doxorubicin-induced cell death was linked to autophagy (i.e., autophagy-associated cell death).

**Figure 4:**
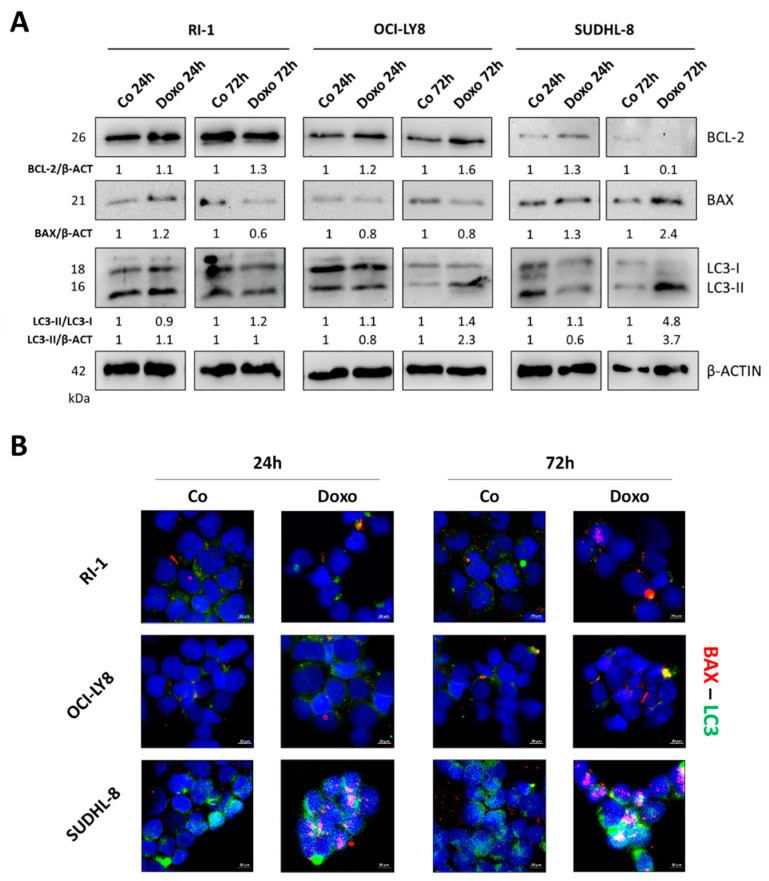
Assessment of involvement of autophagy in doxorubicin-promoted cell death. RI-1, OCI-LY8 and SUDHL-8 cells were treated with doxorubicin for 24 h and 72 h. (**A**) Western blotting analysis of the expression of BAX, BCL-2 and LC3. The filters were probed with β-actin as a loading control. Densitometric data are reported. (**B**) Immunofluorescence double staining BAX (red)/LC3 (green). Scale bar = 20 µm; magnification = 63×. The data shown in this figure are representative of three independent experiments that showed a similar trend.

As shown in Figure 4A, RI-1 and OCI-LY8 cells treated with doxorubicin did not display gross modulation of autophagy flux (the LC3-II/LC3-I ratio did not or only slightly change during incubation) or of the BAX levels during the studied time period. A marked increase in the BAX level along with the strong induction of autophagy (indicated by the increase in the LC3-II/LC3-I ratio compared to that of the control) was instead observed in SUDHL-8 doxorubicin-treated cells at 72 h. Notably, these cells displayed the lowest BCL-2 expression (Figure 4A) and the highest BECLIN-1 expression (Figure 2A) and highest autophagy flux (Figure 2B), which strongly suggest a pivotal role of autophagy in the chemosensitivity to doxorubicin treatment.

To test the hypothesis that doxorubicin-induced cell death is in fact associated with exacerbated autophagy, we performed immunofluorescence double staining for BAX and LC3, which, respectively, marked the apoptotic and autophagy-positive cells (Figure 4B). RI-1 and OCI-LY8 showed a cytoplasmic diffuse LC3 fluorescent signal, indicating the prevalence of the immature LC3-I isoform compared to the vacuolar LC3-II isoform. The treatment with doxorubicin did not elicit significant changes in LC3 and BAX levels in both cell lines. On the other hand, SUDHL-8 cells exposed to doxorubicin displayed a marked increase in BAX expression and, more importantly, the BAX-positive cells also exhibited a high LC3 signal, which was consistent with our hypothesis that the sensitivity of SUDHL-8 cells was due to autophagy-associated cell death.

### 3.3. BCL2 Is a Negative Prognostic Factor while High BECN1 Expression Predicts Better Outcomes for DLBCL Patients

**Figure 5:**
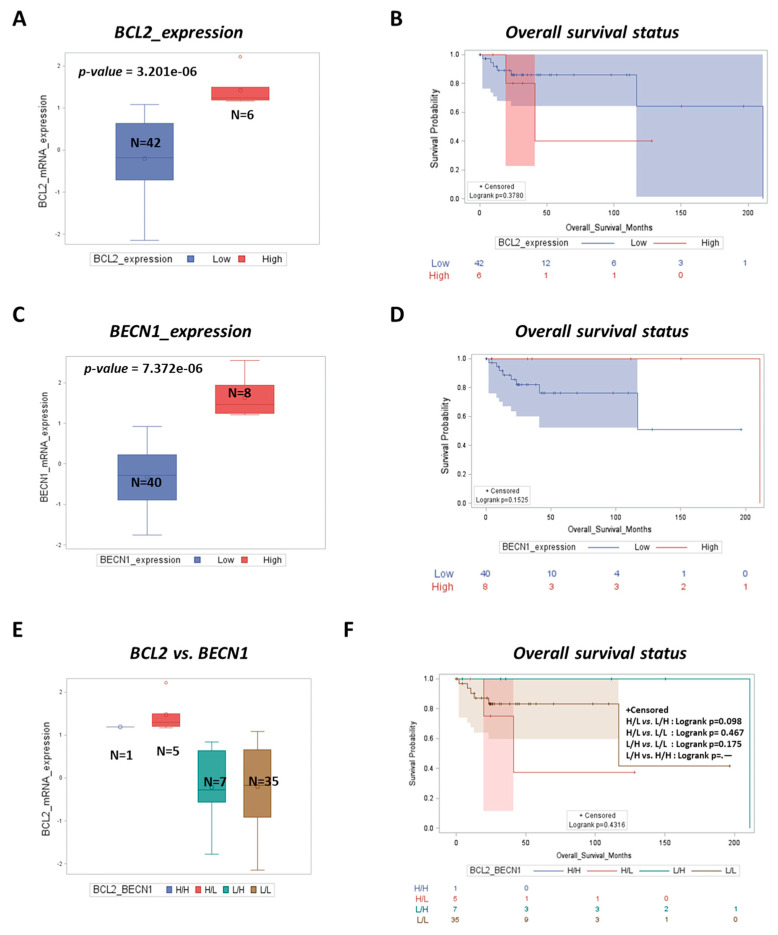
High *BECN1* mRNA expression with low *BCL2* mRNA expression associated with longer overall survival. (**A**) Boxplot showing the distribution of *BCL2* expression based on expression levels (high vs. low). (**B**) Overall survival status for DLBCL patients based on *BCL2* expression levels (high vs. low). (**C**) Boxplot showing the distribution of *BECN1* expression based on expression levels (high vs. low). (**D**) Overall survival status for DLBCL patients based on *BECN1* expression levels (high vs. low). (**E**) Boxplot showing the distribution of *BCL2* expression based on *BCL2* and *BECN1* expression levels (H/H, H/L, L/H, and L/L). (**F**) Kaplan–Meier plot representing the overall survival status of DLBCL patients stratified on the basis of differential expression of *BCL2* and *BECN1* expression levels ((H/H, H/L, L/H, and L/L).

To address the translational relevance of the above findings, we tested the prognostic roles of the *BCL2* and *BECN1* genes in the TCGA DLBCL cohort (Figure 5). We found that patients with high *BCL2* expression (*p* = 3.201 × 10^−6^) displayed a poor prognosis (*p* = 0.3780) compared to those with low *BCL2* expression (Figure 5A,B). Notably, overall survival (*p* = 0.1525) was higher in patients bearing a tumor with high *BECN1* expression (*p* = 7.372 × 10^−6^) than in those with low *BECN1* expression (Figure 5C,D).

Next, we performed a correlation analysis of the above genes and then we classified the patients into sub-groups based on the level of co-expression of both *BCL2* and *BECN1*. Patients were sub-divided into four groups, namely High/High (H/H), High/Low (H/L), Low/High (L/H), and Low/Low (L/L), based on the expression of *BCL2* and *BECN1*, respectively. Multiple variable survival analysis showed that low *BCL2* expressor patients with high *BECN1* expression (L/H) had longer overall survival (*p* = 0.098) (Figure 5E,F) compared to the other groups (L/L, H/L, and H/H), which were associated with a poor prognosis. Taken together, these data suggest that the low expression of *BCL2* and high *BECN1* expression result in a marked improvement in clinical outcomes, highlighting that *BECN1* may represent a positive prognostic marker for DLBCL.

To assess the biological processes involved in the differential outcomes described above, we performed an in-silico transcriptomic analysis on the DLBCL dataset. We retrieved RNA-seq data (mRNA expression profile) from the TCGA database and performed a co-expression analysis to identify the most significant differentially expressed genes (DEGs) correlated with *BCL2* and *BECN1* expression. We found that *BECN1* negatively correlated genes (corresponding to *BCL2* positively correlated transcripts) were involved in the regulation of proliferation and survival pathways and of the cell cycle, while *BECN1* positively correlated genes (corresponding to *BCL2* negatively correlated transcripts) were associated with apoptosis and autophagy lysosomal proteolysis (Supplementary Figures S1 and S2).

The patients were divided into two groups based on the differential expression of *BCL2* and *BECN1*. Based on their expression patterns, we selected five patients for each group as follows: (i) Group A included patients with high *BECN1* expression and low *BCL2* expression, and (ii) Group B included patients with high *BCL2* expression together with low *BECN1* expression. The most significant DEGs were screened and selected for each biological process and pathway from the ones identified in the Gene Ontology reported in Supplementary Figures S1 and S2.

**Figure 6:**
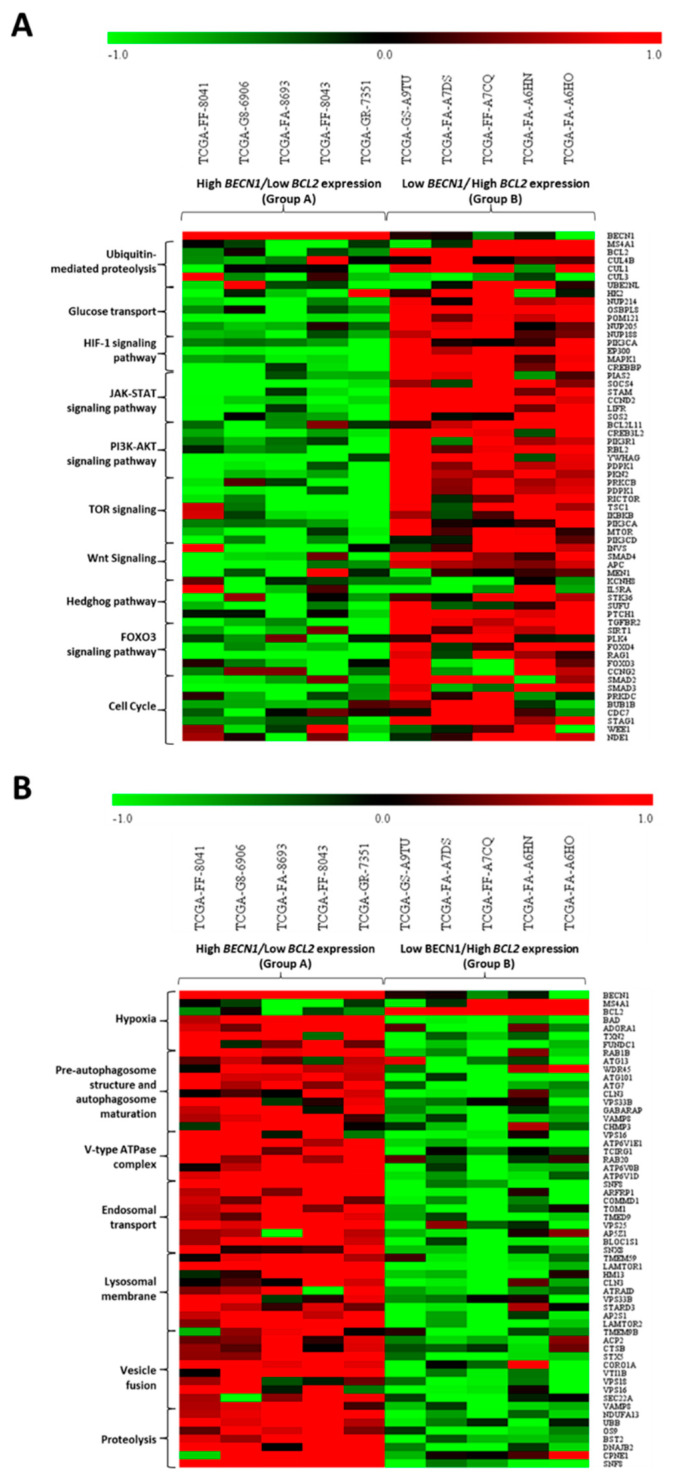
Comparison of differentially expressed genes in two groups of patients stratified based on *BECN1* and *BCL2* expression. Patients were stratified on the basis of high *BECN1* expression (Group A) and high *BCL2* expression (Group B). Heatmaps showing the top 8 genes related to biological processes related to oncogenic pathways (**A**) and autophagy lysosomal proteolysis (**B**).

From the heatmaps reported in Figure 6, one can appreciate that patients of Group A (showing a better prognosis) displayed the downregulation of a range of transcripts involved in oncogenic pathways, including the glucose transport, HIF1A, PI3K-AKT, Wnt, JAK-STAT, mTOR, and Hedgehog signaling pathways and the cell cycle (Figure 6A), in parallel with the upregulation of transcripts belonging to cell death and autophagy lysosomal proteolysis (Figure 6B). In contrast, patients belonging to Group B (associated with poor prognosis) displayed the opposite trend compared to that of Group A, displaying the upregulation of oncogenic pathways along with the downregulation of genes involved in the regulation of autophagy.

Taken together, the above data strongly support the hypothesis that the clinical outcome in DLBCL patients is strictly correlated with BECLIN-1-dependent autophagy, which is antagonized by BCL-2. In the next section, we report our experiments in DLBCL cell lines, where we tested whether the manipulation of BECLIN-1 or BCL-2 expression and function effectively impact doxorubicin sensitivity and autophagy-associated cell death.

### 3.4. Spautin-1-Mediated Inhibition of BECLIN1-Dependent Autophagy Reduces the Sensitivity of SUDHL-8 Cells to Doxorubicin Treatment

**Figure 7:**
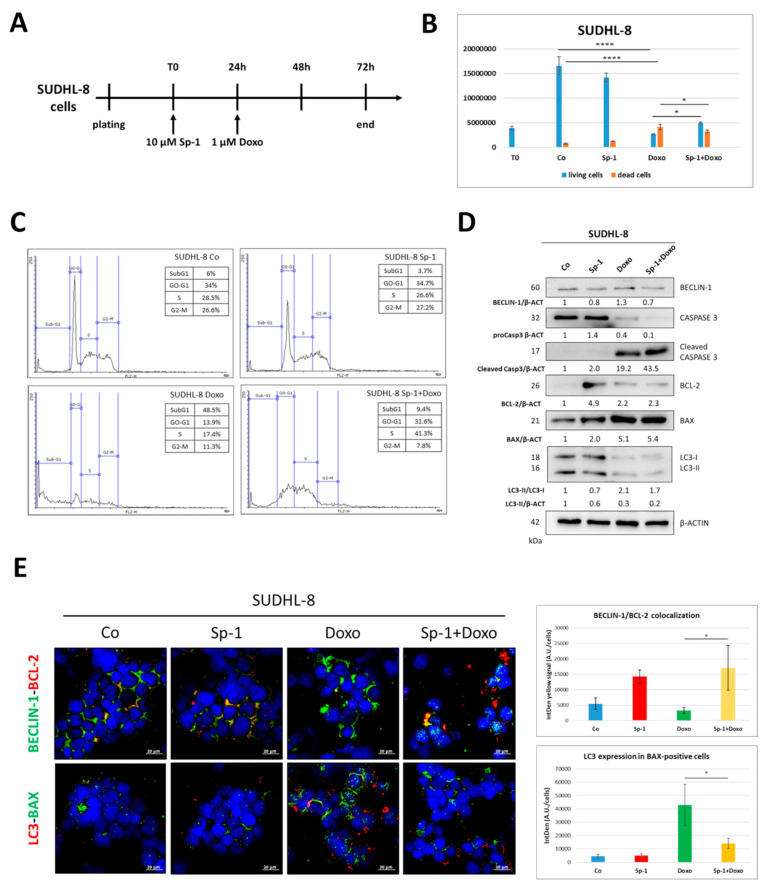
Inhibition of BECLIN-1-dependent autophagy reduces the sensitivity of SUDHL-8 cells to doxorubicin. (**A**) Experimental timeline. SUDHL-8 cells were pre-treated with spautin-1 for 24 h, and then doxorubicin was added and cells were cultured for a further 48 h. (**B**) Cell counting by Trypan blue exclusion was performed in triplicate. Significance was considered as follows: **** *p* < 0.0001; * *p* < 0.05. (**C**) Cell cycle analysis was performed using a FacScan flow cytometer. (**D**) Assessment of apoptosis (BAX and caspase 3 cleavage) and autophagy marker (BECLIN-1 and LC3) expression by Western blotting. Densitometric data are reported. (**E**) Immunofluorescence double staining BECLIN-1 (green)/BCL-2 (red) and BAX (green)/LC3 (red). Scale bar = 20 µm; magnification = 63×. Graphs report the quantification of BECLIN-1/BCL-2 colocalization and LC3 levels in BAX-positive cells. Significance was considered as follows: * *p* < 0.05.

First, we tested whether the sensitivity of SUDHL-8 cells to doxorubicin is indeed associated with BECLIN-1-dependent autophagic cell death. To this end, the cells were pre-treated with spautin-1, which causes the proteasome degradation of BECLIN-1 [27], followed by doxorubicin treatment. The details of the experimental timeline are reported in Figure 7A.

Figure 7B shows that the pre-treatment with spautin-1 reduced the number of dead cells compared to that in doxorubicin-only-treated cells. Moreover, the cytofluorimetric analysis showed that co-treated cells exhibited a significantly lower SubG1 fraction compared to that of doxorubicin-only-treated cells (SubG1 = 9.4% vs. 48.5%, respectively), and these cells tended to display cell cycle arrest in the S phase (Figure 7C). Finally, we assayed, by Western blotting, the expression of LC3 (autophagy marker) and of BAX and caspase 3 (apoptotic markers) (Figure 7D). Spautin-1-treated cells displayed a decrease in BECLIN-1 levels, confirming that the treatment was effective, and in the LC3-II/LC3-I conversion rate, consistently indicating the inhibition of autophagy. Notably, spautin-1 and doxorubicin co-treated cells displayed a lower fraction of cleaved caspase 3 and a reduction in BAX levels compared to doxorubicin-only-treated cells, confirming that the inhibition of BECLIN-1-dependent autophagy decreases doxorubicin-induced cell death. These results were further supported by immunofluorescence double staining for BCL-2/BECLIN-1 and BAX/LC3 (Figure 7E), which showed the increased colocalization of BCL-2/BECLIN-1 in parallel to a reduction in LC3 and BAX expression, indicating the downregulation of autophagy-associated cell death in co-treated cells compared to doxorubicin-only-treated cells.

### 3.5. Venetoclax-Mediated Targeting of BCL-2 Sensitizes RI-1 Cells to Doxorubicin Treatment

**Figure 8:**
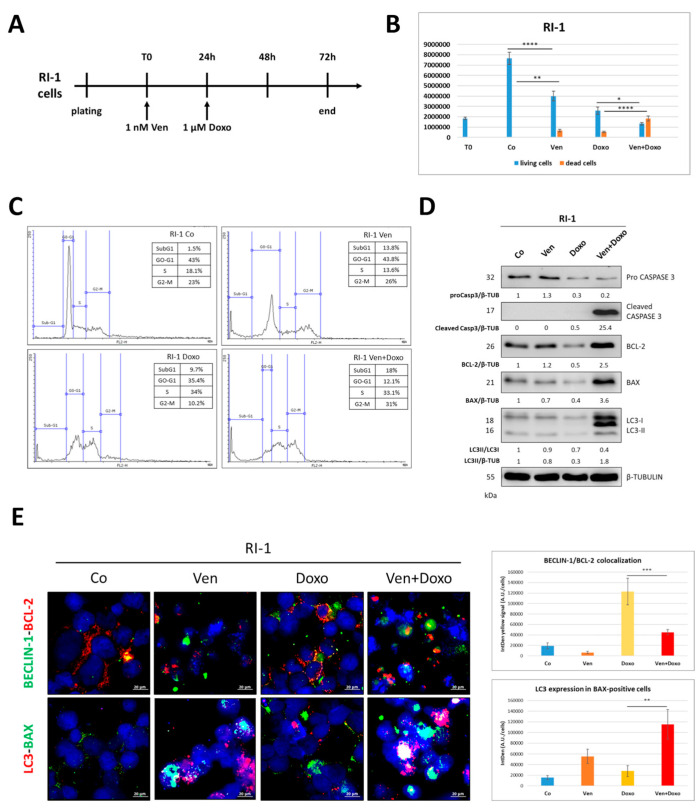
Targeting BCL-2 sensitizes RI-1 cells to doxorubicin treatment. (**A**) Experimental timeline. RI-1 cells were pre-treated with venetoclax for 24 h, and then doxorubicin was added, and cells were cultured for a further 48 h. (**B**) Cell counting by Trypan blue exclusion was performed in triplicate. Significance was considered as follows: **** *p* < 0.0001; ** *p* < 0.01; * *p* < 0.05. (**C**) Cell cycle analysis was performed using a FacScan flow cytometer. (**D**) Assessment of apoptosis (BAX and caspase 3 cleavage) and autophagy marker (LC3) expression by Western blotting. (**E**) Immunofluorescence double staining BECLIN-1 (green)/BCL-2 (red) and BAX (green)/LC3 (red). Scale bar = 20 µm; magnification = 63×. Graphs report the quantification of BECLIN-1/BCL-2 colocalization and LC3 levels in BAX-positive cells. Significance was considered as follows: *** *p* < 0.001; ** *p* < 0.01.

RI-1 cells were shown to express the highest levels of BCL-2 along with low levels of BECLIN-1 expression and low autophagy flux (Figure 2). To test whether antagonizing BCL-2 can sensitize RI-1 cells to chemotherapy via enhancing autophagy, we pre-treated these cells with venetoclax (a chemotherapeutic drug targeting BCL-2), followed by exposure to doxorubicin. The details of the experimental timeline are reported in Figure 8A. We monitored the cytotoxic effects of doxorubicin by cell counting, which showed a slight increase in dead cells in venetoclax-only-treated cells, similar to that observed in doxorubicin-only-treated cells, while we observed a great increase in cell death with the co-treatment (Figure 7B). Consistently, the cytofluorometric cell cycle analysis (Figure 7C) showed that the pre-treatment with venetoclax sensitized RI-1 cells to doxorubicin, as indicated by the higher SubG1 fraction compared to that of doxorubicin-only-treated cells (SubG1 = 18% vs. 9.7%, respectively). Additionally, we noticed that venetoclax alone elicited an increase in cell death (SubG1 = 13.8%), pointing to the prominent role of BCL-2 for the survival of these cells. To test whether antagonizing BCL-2 could result in the restoration of autophagy, which in turn could sensitize RI-1 cells to chemotherapy, we monitored the expression of apoptotic markers and autophagy flux by Western blotting (Figure 7D). Compared to doxorubicin alone, the co-treatment induced a marked increase in BAX and the cleaved caspase 3 fraction in parallel with a decrease in BCL-2 and pro-caspase 3 expression (which was barely detectable). To confirm whether these effects were mechanistically linked with autophagy, we performed two immunofluorescence co-staining procedures for BECLIN-1/BCL-2 and LC3/BAX (Figure 7E). In venetoclax-treated cells (either challenged or not with doxorubicin), we detected decreased colocalization of BECLIN-1/BCL-2 paralleled by the upregulation of LC3-positive spots and BAX levels, indicating that the pre-treatment with venetoclax was synergized with doxorubicin by restoring autophagy.

## 4. Discussion

DLBCL represents approximately 30% to 40% of B-NHLs and shows a heterogeneous clinical presentation that reflects the molecular and cellular heterogeneity of the disease. Accordingly, the clinical outcome of DLBCL is extremely variable, with 60% to 70% of patients responding to chemoimmunotherapy and the remaining 30 to 40% of patients showing resistance or relapse, determining a poor prognosis [32]. The stratification of DLBCL in terms of poor and good responders to the R-CHOP standard regimen is crucial for the implementation of personalized B-cell lymphoma treatment, and therefore it is important to genotype the tumor to identify the critical risk-associated genes [16,33,34]. In this respect, high-throughput DNA sequencing has provided useful information [35]. However, this information has not been translated yet into novel approaches for improved treatment. Investigations of the genetic alterations and the molecular pathways linked to relapse and resistance to chemoimmunotherapy are therefore needed.

Autophagy is a well-studied homeostatic pathway that is involved in cellular metabolism, the regulation of cell death, and immune responses, and its deregulation results in lymphoma development and correlates with poor clinical outcomes [19,24,36]. Nonetheless, the mechanistic and prognostic roles of autophagy in DLBCL have been poorly explored. Depending on the cellular context, autophagy suppresses tumorigenesis by removing damaged organelles/proteins, limits cell growth, and its hyper-induction can sensitize cancer cells to chemotherapy [28,37]. In other contexts, it may also provide energy to tumor cells in nutrition-lacking microenvironments and acts as an adaptive cellular process in anticancer therapy, supporting tumor cell survival and drug resistance [28,38].

In the present work, we focused on *BECN1*, a tumor suppressor gene that positively regulates autophagy, and tested its prognostic value, either alone or in combination with *BCL2*, as a marker for DLBCL patient risk stratification. Our laboratory previously investigated the potential link between BECLIN-1-mediated autophagy and the clinical outcome of B-NHLs. We reported that B-NHLs in which ≥20% of tumor cells expressed high levels of BECLIN-1 were associated with complete (57%) or partial (35%) remission. These data prompted us to hypothesize that B-NHLs with upregulated autophagy are more responsive to chemotherapy and indicated that BECLIN-1 could be a valuable independent prognostic factor in this heterogeneous group of tumors [24].

*BCL2* codes for an oncogenic anti-apoptotic protein that is often hyper-expressed in DLBCL, especially of the GCB subtype, because of chromosomal translocation t(14;18) (q32;q21) [39]. Activating mutations in its promoter [10,40,41,42] or gene amplification [30,41,42,43] have also been reported. In the TCGA database, six patients bore a tumor with high expression of *BCL2* mRNA, four of which carried a gene amplification. It has been reported that patients harboring *BCL2* overexpression have remarkably inferior 5-year progression-free survival and poor overall survival upon R-CHOP therapy, while mutations and translocations are not significantly prognostic for survival [44,45]. It must be stressed that the BCL-2 protein is an interactor with the BECLIN-1 protein [20], and when these two proteins are bound to each other, pro-survival autophagy is impaired while apoptosis is permitted [21,46]. Thus, the relative expression of *BCL2* and *BECN1*, and the availability of the respective proteins, will determine the fate of the cell in response to the chemoimmunotherapy-induced cell toxicity. Here, we report that in the TCGA cohort, the expression of *BECN1* is significantly negatively correlated with that of *BCL2*.

To mechanistically prove that BECLIN-1-dependent autophagy may sensitize DLBCL cells to chemotherapy, we chose three DLBCL cell lines with different genetic backgrounds of *BECN1* and *BCL2* and we compared their responses to doxorubicin-induced cell toxicity. We observed that highly BCL-2-expressing cells (RI-1 and OCI-LY8) treated with doxorubicin did not significantly modulate autophagy flux and apoptosis, indicating that, in these cells, the effect was mainly cytostatic. In contrast, SUDHL-8 cells that expressed high levels of BECLIN-1 and showed a high level of autophagy flux displayed autophagy-associated cell toxicity when challenged with doxorubicin. Consistent with this interpretation, we found that the spautin-1-mediated abrogation of BECLIN-1-dependent autophagy resulted in the decreased sensitivity of SUDHL-8 cells to doxorubicin. Specifically, targeting BCL-2 with venetoclax restores autophagy flux and renders RI-1 cells more susceptible to doxorubicin-induced cell death.

These findings are in line with the report of Brem and colleagues, who stated that obatoclax, a BH3 mimetic that can specifically target and counteract Bcl-2 family proteins, results in increased cell death via an autophagy-mediated pathway in rituximab-resistant DLBCL cells [47]. Additionally, the inhibition of autophagy by 3-methyladenine or by siRNA knockdown of BECLIN-1 diminished the anti-tumor activity of obatoclax in DLBCL cells [47]. However, given the complex role of autophagy in cancer, these findings should be always considered in the specific context. In contrast to our findings, other studies have reported that the inhibition of the classical autophagy pathway by tenovin-6, an inhibitor of sirtuin 1, or by knocking down key genes in the autophagy pathway, impairs the proliferation and survival of DLBCL cells [48]. Another study conducted by Rosich and colleagues on mantle cell lymphoma (MCL) revealed that autophagy was upregulated in MCL cells refractory to a combinatory treatment with everolimus (an mTOR inhibitor) and an Akt inhibitor. Accordingly, knockdown of the autophagy genes *ATG7*, *ATG5*, and *ATG3*, and pre-treatment with the autophagy inhibitor hydroxychloroquine, efficiently overcame the resistance to Akt/mTOR inhibitors, leading to the activation of the mitochondrial apoptotic pathway [49].

To address the translational impact of our study, we performed a large meta-analysis on the transcriptomic data of DLBCL patients to identify targets in relation to the *BECN1* and *BCL2* genes that together determine the cell fate in response to chemotherapy through governing pathways such as autophagy, cell survival, and cell death. Our analysis showed that patients with low expression of *BECN1* and high *BCL2* expression exhibited reduced overall survival (although the differences were not statistically significant because of the low number of patients) compared to those expressing low *BCL2* along with high *BECN1*. The transcriptomic analysis revealed that the DEGs belonging to *BECN1* and *BCL2* expression were inversely correlated. Based on this analysis, the high expression of *BECN1* was associated with the inhibition of B-cell survival and their proliferation by downregulating pathways involved in cell morphogenesis, cell growth, cell survival (MAPK, Wnt, JAK-STAT, mTOR, TGF-β pathways), the cell cycle, and glucose transport, while upregulating the transcripts belonging to proteolysis, vesicle fusion, and endosomal transport. However, the present work had one limitation related to the small number of TCGA DLBCL patients (N = 48), which allowed us only to describe the trends, without reaching statistical significance. In the future, we aim to extend our bioinformatic analysis to other databases with a larger number of cases.

Taken together, our data indicate that autophagy modulation significantly affects the outcome of chemotherapy, and we conclude that BECLIN-1-dependent autophagy is associated with improved therapeutic efficacy in patients with DLBCL, strongly supporting BECLIN-1 as a positive prognostic marker.

## 5. Conclusions

**Figure 9:**
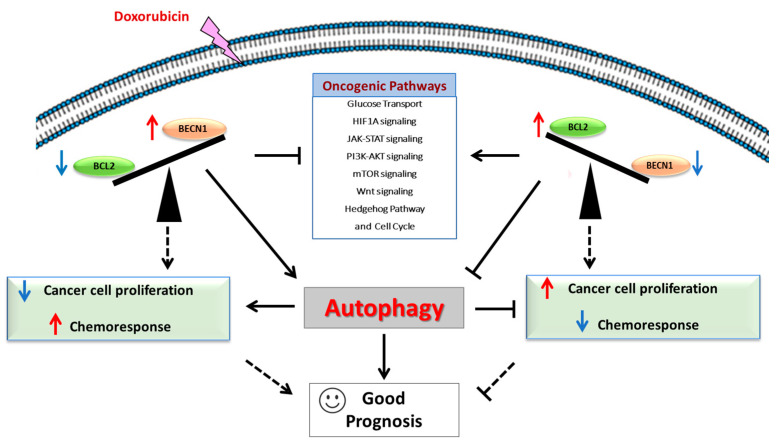
Schematic representation of findings reported in the present study.

To our knowledge, this is the first study investigating the impact of *BECN1* expression on the transcriptomes of DLBCL patients. Our data show that autophagy induction improves clinical outcomes in lymphoma patients, suggesting that autophagy modulation may be a crucial target in overcoming acquired resistance and improving the clinical outcomes of patients undergoing R-CHOP therapy (Figure 9). In detail, promoting BECLIN1-dependent autophagy through the disruption of BECLIN-1/BCL-2 interaction—for instance, with venetoclax—could improve the chemotherapy response in refractory DLBCL, providing insights for the design of novel clinical trials that include autophagy inducers in the clinical management of this tumor. However, it must be stressed that the role of autophagy in tumor progression is contradictory, as it could either promote or counteract chemoresistance [19]. Our study of the transcriptomic profile may provide an answer to this apparent contradiction. A personalized solution would be to profile each patient for the expression of the relevant genes, in order to select those who could truly benefit from a pro-autophagy therapy.

## Figures and Tables

**Table 1:** Mutational status of relevant oncogenes and tumor suppressor genes.

Cell Line	*BCL2*	*BCL6*	*MYC*	*TP53*	*PTEN*	*BECN1*
**RI-1**	Wild type	Wild type	Wild type	Missense mutation	Wild type	Missense mutation
**OCI-LY8**	Wild type	Wild type	Wild type	Wild type	Wild type	Wild type
**SUDHL-8**	Missense mutation	Wild type	Missense mutation	Missense mutation	Wild type	Wild type

## Data Availability

Not applicable.

## Supplementary Materials

The following supporting information can be downloaded at: https://www.mdpi.com/article/10.3390/cells12151924/s1, Supplementary Table S1: Characteristics of lymphoid neoplasm diffuse large B-cell lymphoma patients (TCGA, Firehose Legacy). Supplementary Figure S1: *BCL2* promotes oncogenic process and pathways. (A) Volcano plot displaying the differentially expressed genes (DEGs) based on *BCL2* co-expression. Red dots represent DEGs with Spearman’s correlation value ≥+0.4. Blue dots represent DEGs with Spearman’s correlation value ≥−0.4. The top 300 genes are highlighted and both up- and downregulated genes are reported in the graphs. −Log10 (*p*-value) was fixed above 2.0. (B) Biological processes associated with the top 841 *BCL2* positively correlated genes. (C) Biological processes associated with the top 974 *BCL2* negatively correlated genes. Supplementary Figure S2: *BECN1* inhibits oncogenic process and pathways. (A) Volcano plot displaying the differentially expressed genes (DEGs) based on *BECN1* co-expression. Red dots represent DEGs with Spearman’s correlation value ≥+0.4. Blue dots represent DEGs with Spearman’s correlation value ≥−0.4. The top 300 genes are highlighted and both up- and downregulated genes are reported in the graphs. −Log10 (*p*-value) was fixed above 2.0. (B) Biological processes associated with the top 649 *BECN1* positively correlated genes. (C) Biological processes associated with the top 437 *BECN1* negatively correlated genes.
