# Supplementary material for: High BECN1 Expression Negatively Correlates with BCL2 Expression and Predicts Better Prognosis in Diffuse Large B-Cell Lymphoma: Role of Autophagy
Source: Cells. 2023 Jul 25;12(15):1924. doi: 10.3390/cells12151924 (PMC10417641; doi:10.3390/cells12151924)
Supplement: Supplementary file 1 [file cells-12-01924-s001.zip › Supplementary Table S1 - Clinical charecteristic of DLBCL Patients.pdf]

Supplementary Table S1. Characteristics of Lymphoid Neoplasm Diffuse Large B-cell Lymphoma Patients (TCGA, Firehose Legacy).

Clinical Characteristics	Number	Percentage
Diffuse Large B-cell Lymphoma Patients	48	100
Sex		
Male	22	45.83
Female	26	54.17
Age		
Age range 23-82	57 (median)	
> 60 (median age)	21	43.75
≤ 60	27	56.25
Extranodal sites		
< 2	25	52.1
≥ 2	12	25
N/A	11	22.91
Clinical stage		
III-IV	17	35.41
I-II	25	52.01
N/A	6	12.5
BCL2 status		
Positive	24	50
Negative	8	16.66

N/A	16	33.33
Adjuvant Postoperative Targeted Therapy		
Chemotherapy	22	45.83
Chemotherapy+ Radiotherapy	1	2
N/A	22	45.83
Primary Therapy Outcome		
Complete Response	18	37.5
Partial Response	3	6.25
Progressive Disease	2	4.16
Stable Disease	2	4.16
N/A	22	45.83
