# Supplementary figures and images for: High BECN1 Expression Negatively Correlates with BCL2 Expression and Predicts Better Prognosis in Diffuse Large B-Cell Lymphoma: Role of Autophagy
Source: Cells. 2023 Jul 25;12(15):1924. doi: 10.3390/cells12151924 (PMC10417641; doi:10.3390/cells12151924)

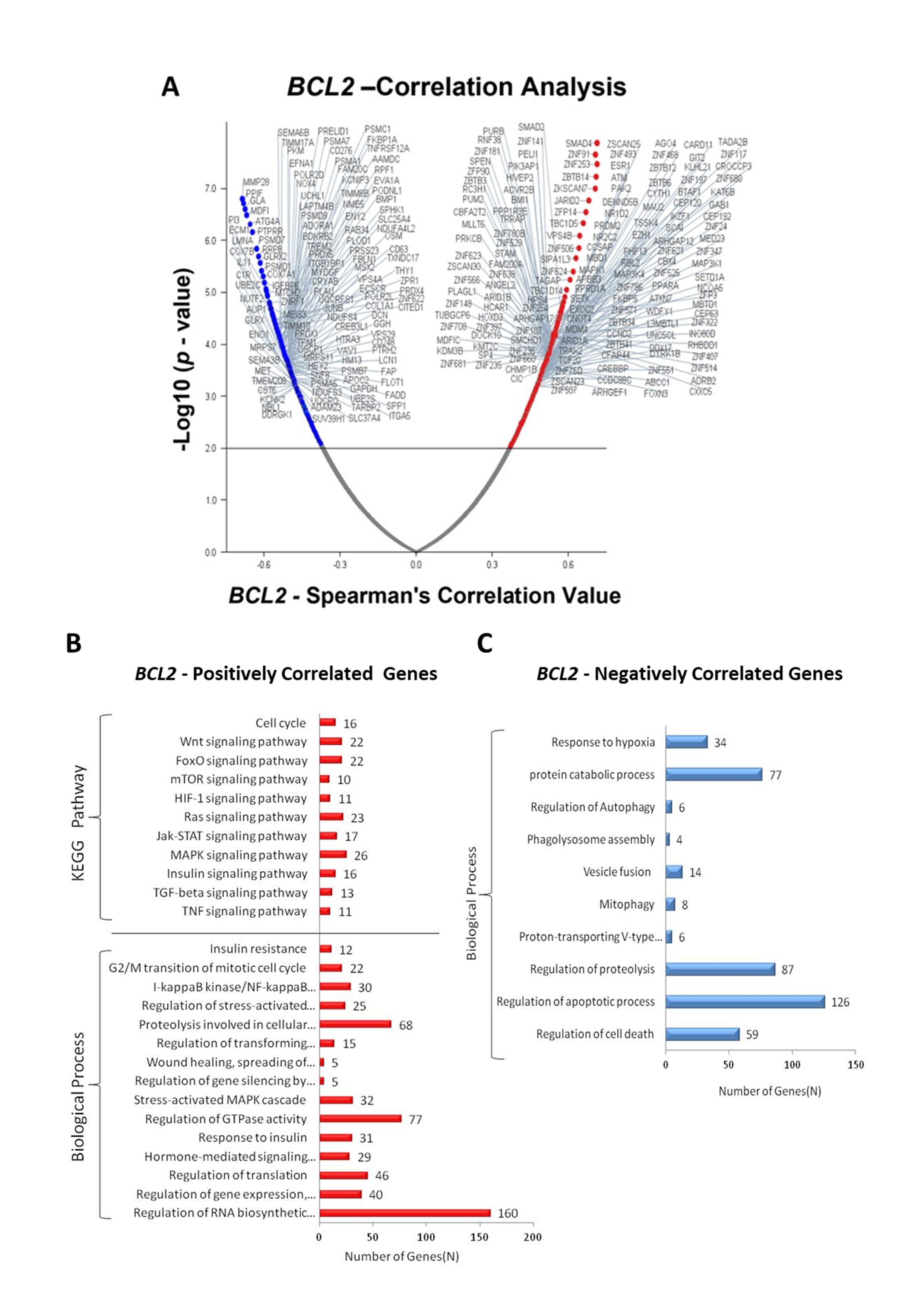

Supplement: Supplementary file 1 [file cells-12-01924-s001.zip › Supplementary Figure S1.tif]

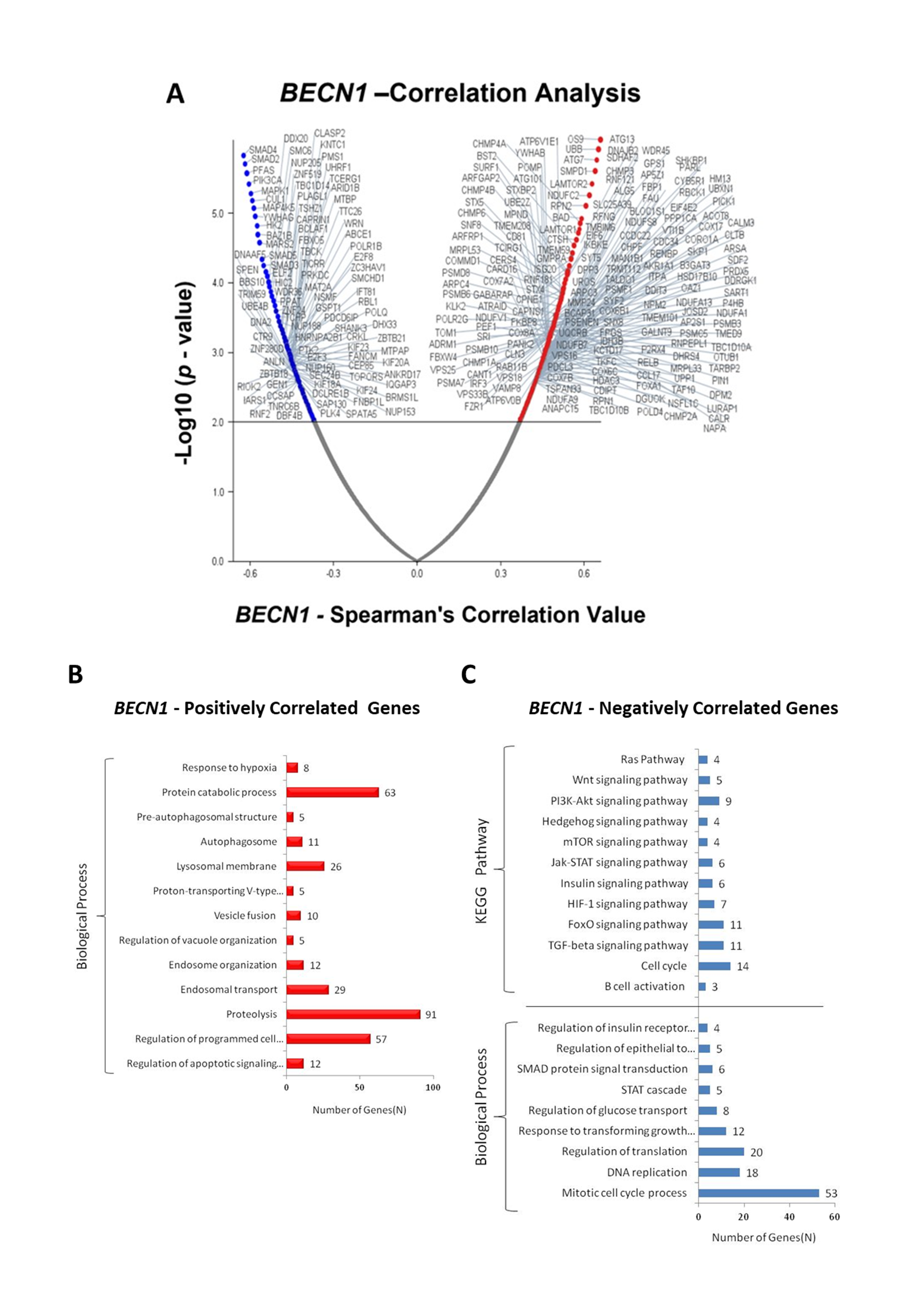

Supplement: Supplementary file 1 [file cells-12-01924-s001.zip › Supplementary Figure S2.tif]
